# Factors mediating the impacts of child abuse and intimate partner violence on chronic pain: a cross-sectional study

**DOI:** 10.1186/s12905-018-0642-9

**Published:** 2018-10-03

**Authors:** Eman Alhalal, Marilyn Ford-Gilboe, Carol Wong, Fadia AlBuhairan

**Affiliations:** 1 Nursing College, King Saud University, Riyadh, Saudi Arabia; 2 Arthur Labatt Family School of Nursing, Western University, London, ON Canada; 3 Department of Pediatrics and Adolescent Medicine, AlDara Hospital and Medical Center, Riyadh, Saudi Arabia; 4 Department of Population, Family, and Reproductive Health Bloomberg School of Public Health, Johns Hopkins University, Baltimore, MD USA

**Keywords:** Intimate partner violence, Child abuse, Depressive symptoms, Post-traumatic stress disorder symptoms, Chronic pain, And social support

## Abstract

**Background:**

Most research on the health impacts of intimate partner violence (IPV) and child abuse has been conducted in Western countries and may not be generalizable to women living in different contexts, such as Saudi Arabia. Chronic pain, a disabling health issue associated with experiences of both child abuse and IPV among women, negatively impacts women’s well-being, quality of life, and level of functioning. Yet, the psychosocial mechanisms that explain how abuse relates to chronic pain are poorly understood. We developed and tested a theoretical model that explains how both IPV and child abuse are related to chronic pain.

**Methods:**

We recruited a convenience sample of 299 Saudi women, who had experienced IPV in the past 12 months, from nine primary health care centers in Saudi Arabia between June and August 2015. Women completed a structured interview comprised of self-report measures of IPV, child abuse, PTSD, depressive symptoms, chronic pain, and social support. Using Structural equation modeling (SEM), we analyzed the proposed model twice with different mental health indicators as mediators: PTSD symptoms (Model 1) and depressive symptoms (Model 2).

**Results:**

Both models were found to fit the data, accounting for 31.6% (Model 1) and 32.4% (Model 2) of the variance in chronic pain severity. In both models, mental health problems (PTSD and depressive symptoms) fully mediated the relationship between severity of IPV and child abuse and chronic pain severity. Perceived family support partially mediated the relationship between abuse severity and depressive symptoms.

**Conclusions:**

These results underscore the significance of considering lifetime abuse, women’s mental health (depressive and PTSD symptoms) and their social resources in chronic pain management and treatment.

## Background

Violence against women is a major social and public health problem worldwide [1]. Women and girls are at increased risk of multiple forms of violence including intimate partner violence (IPV), child abuse, sexual violence during civil conflict, and trafficking [2, 3]. IPV is a pattern of physical, emotional, and/or sexual abuse by a current or former intimate partner in the context of coercive control [4]. IPV has been linked to an extensive array of health problems, including traumatic brain injury, migraines, fatigue, post-traumatic stress disorder (PTSD), and depression [5, 6]. Abused women make more health care system visits than women in the general population [1], resulting in increased costs [7]. There is evidence that experiencing child abuse increases the risk of IPV victimization and perpetration in adulthood [8, 9] and leads to various negative health conditions [10]. Research has highlighted how experiencing both child abuse and IPV leads to poorer health in comparison with experiencing a single form of abuse, providing support for the position that the effects of abuse are cumulative [11, 12]. Yet, women’s health has typically been studied primarily as a consequence of recent IPV experiences without sufficiently considering the interactive effects of previous abuse experiences [13].

Chronic pain, defined as pain that persists for longer than three months or beyond the time of usual healing [14], is a highly disabling health problem that has been linked to both IPV [15, 16] and a history of child abuse [17]. The prevalence of chronic pain among women who have experienced IPV have been found to range from 38 to 94.5% [18–21]. The negative consequences of living with chronic pain include depleted emotional reserves [22], lower quality of life [23], poorer interpersonal and family functioning [24], and decreased productivity [25]. Disability related to chronic pain is common among abused women and limits their ability to carry out important social roles and lead satisfying lives [20, 26]. At the system level, chronic pain is costly as a result of healthcare utilization [27].

Chronic pain is thought to result from the complex and dynamic interplay of physiological, psychological and social factors [25, 26]. Exposure to chronic stressors or trauma have been found to result in prolonged activation of the stress system leading to allostatic load [27]. A possible consequence of allostatic load is dysfunction of hypothalamic-pituitary-adrenal (HPA) axis [28] which induces alterations in cortisol level [29] and pro-inflammatory cytokine levels [30] and leads to deficiencies in tissue repair, impairment in immune system, atrophy of muscle tissue, and excessive inflammation observed in chronic pain [29, 31]. In the context of IPV, chronic pain can be understood as a biopsychosocial response to *abuse*. Specifically, among women experiencing IPV, chronic pain may result from neuropathic alterations associated with abuse related-injuries [32] and/or to HPA axis dysfunction [33], abnormal levels of cortisol [34], and alterations in pro-inflammatory cytokine levels [35] previously described that are consequences of living with both trauma and the chronic stress associated with IPV. Research on the physiological origins of chronic pain among IPV survivors has generated important insights, yet the complex psychosocial pathways that connect severity of abuse with chronic pain are still poorly understood. Specifically, knowledge about modifiable psychosocial mediators of the relationship between severity of abuse and chronic pain could inform clinical interventions to better manage chronic pain or reduce its disabling impacts for women. Moreover, most of existing evidence is based on research in Western countries and may not be generalizable to women living in different socio-cultural contexts.

Women’s mental health and access to social support may be important mediators of the relationship between experiences of abuse and severity of chronic pain, yet there are substantial gaps in knowledge about whether and how these factors acts as mediators. Depression and PTSD are common mental health consequences of both IPV and past child abuse experiences [36–38]. Research has documented the co-occurrence of chronic pain with both PTSD [39] and depression [40] in various populations, including women with histories of IPV [41, 42]. Depression has been identified as a risk factor for the onset of chronic pain and disability-related to pain [40, 43] such that higher levels of depressive symptoms are associated with more severe chronic pain [44]. Among IPV survivors, PTSD symptoms have been found to predict chronic pain [45] and to mediate the relationships between both a history of child abuse [46] and chronic pain, and IPV and chronic pain [47]. Few studies have examined whether depressive symptoms mediate the impact of IPV on chronic pain, and these studies have produced inconsistent findings. While Wuest and colleagues [48] found that depressive symptoms significantly mediated the relationships between both child abuse and IPV and chronic pain among Canadian women, no evidence of mediation was found between IPV and chronic pain among Chinese women [16].

Whether symptoms of depression and PTSD mediate the impact of abuse on chronic pain in similar or different ways is unclear. Although PTSD and depression often co-occur in people who have experienced trauma [49], they are generally considered to be distinct reactions to traumatic events [50, 51]. However, some have argued that co-occurring PTSD and depression is a single traumatic stress construct [52, 53]. Studies that test whether depressive and PTSD symptoms mediate the relationship between abuse and chronic pain in the same sample, and compare the strength of mediation, could contribute unique evidence to this ongoing debate.

Social support, the perception that emotional support and/or practical assistance is available from family members and friends [44], is an important determinant of health that may also mediate the pathway between abuse and chronic pain. IPV often decreases women’s access to social support because abusive partners intentionally isolate women [54] and women may distance themselves from their social networks due to feelings of shame, humiliation, and guilt [55, 56]. Abused women’s perceptions of social support have been negatively associated with a wide range of health problems, including symptoms of PTSD [57] and depression [58]. A history of child abuse has been associated with poorer perceived social support in adulthood [59] and, consequently to the development of health problems among women [60]. Social support has been found to mediate the relationship between abuse experiences and symptoms of PTSD [61], depression [62, 63], somatic complains [64], and general mental and physical health [65], but some studies have failed to find support for mediation [66, 67]. Whether social support mediates the impact of child abuse and IPV on chronic pain has not been examined. In addition, the distinction between support from family members and friends is considered to be imperative, as different populations depend on different sources of support [68]. For example, in Saudi society, family members are the main providers of social support [69]. Furthermore, the impacts of support from family members versus friends may differ.

Although the body of research on the health consequences of IPV is substantial, it might not applicable to Saudi women. Indeed, whether the health consequences of IPV vary cross-culturally has not been well studied. Given that IPV is shaped by the social context in which it occurs, research must be conducted in ways that take these different contexts into account [70]. The socio-cultural context that shapes child abuse, IPV and women’s health in Saudi Arabia might differ from Western countries, and these differences could lead to variations in health outcomes for women. In the few studies [71–73] examining the health outcomes of IPV in Saudi Arabia, researchers have not used well-established measures or tested the mechanisms by which abuse leads to these health issues. In health care settings, there remains a strong focus on treating physical injuries associated with abuse [74] with limited attention given to the broader mental and physical health impacts of abuse as manifestations of trauma and chronic stress. Evidence about the nature and impacts of IPV among women could increase recognition of IPV as a public health matter that needs action on multiple levels. The lack of a comprehensive understanding about IPV and its outcomes in the Saudi context might lead to health services that undermine women’s health.

**Fig. 1:**
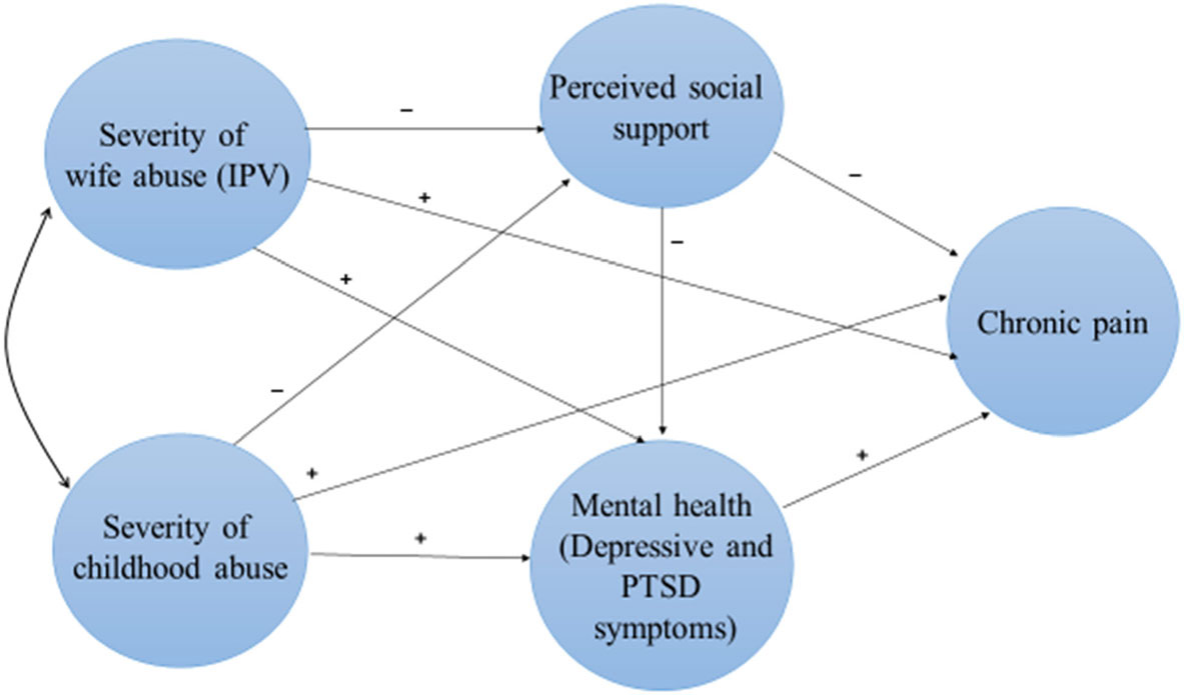
Hypothesized structural equation model derived from the Stress Process Model

To address these gaps, the study aimed to identify the direct and indirect pathways linking child abuse and IPV to chronic pain severity among Saudi women experiencing IPV. We developed a conceptual model informed by Pearlin’s Stress Process Model (SPM), a longstanding sociological framework that addresses how chronic stress impacts health and factors that mediate this process [75]. A shown in Fig. 1, we hypothesized that severity of both IPV and child abuse would affect chronic pain severity directly and, indirectly, through their effects on women’s mental health problems (PTSD and depressive symptoms) and perceived family support (mediators in the model). Specifically, we proposed that more severe child abuse and IPV are related to increased mental health problems (PTSD and depressive symptoms), and to lower perceived family support, each of which are related to more severe chronic pain. We also hypothesized that perceived family support would affect chronic pain indirectly through its impact on mental health problems.

## Method

A cross-sectional, predictive observational study was conducted to examine the complex relationships between severity of child abuse, IPV severity, perceived family support, PTSD symptoms, depressive symptoms and chronic pain severity. To test whether PTSD and depressive symptoms are distinct reactions to traumatic events (i.e. child abuse, IPV), the model was analyzed twice with different mental health variables (PTSD symptoms in Model 1, depressive symptoms in Model 2). Symptoms of PTSD and depression were not included as one latent variable because these mental health issues have different profiles [50, 76].

### Setting and sample

Between June and August 2015, we recruited a convenience sample of married Saudi women, who were between the ages of 18 and 64 years and had experienced IPV in the past 12 months. We screened 774 women for IPV and 311 (40.2%) of those screened had a positive result. Six of 311 women who screened positive for abuse were not willing to participate, resulting in 305 women (98% of those eligible) who agreed to participate. We excluded six women, four of these with missing data ranging from 20.7 to 71.4% on the main variables and two cases in which women answered ‘never’ to all items on the measure of abuse used in this study, despite having screened positive for IPV. Thus, the total sample for the study was 299 women. The sample size was based on the use of structural equation modeling (SEM) for the statistical analysis. According to Kline [77], a minimum sample of 200 is needed to test simple models using SEM, with more complex models requiring larger samples to produce stable estimate estimates and, therefore, more accurate results [78].

The study took place in nine primary health care centers (PHC) in Saudi Arabia. Eight of these centers were in the Eastern Province and one center was located in the capital city Riyadh. These centers provide free curative, preventive, promotive and rehabilitation services including maternal and child health, immunizations, management of chronic diseases (e.g. hypertension and diabetes), provision of essential drugs, and health education. Women were initially asked about their interested in taking part in a women’s health study by a member of the health care team. A member of the research team screened those who were interested for eligibility in a private clinic room. Exposure to IPV was confirmed using the *Abuse Assessment Screen* (AAS) [79], which has been widely used and validated in many settings with pregnant and non-pregnant women [65, 80–82] and also translated to various languages [83, 84]. In this study, the Arabic version of AAS [85] was used as it has a good evidence of reliability [86, 87], but was adapted to be used with non-pregnant women. It included four items capturing physical abuse, forced sex, fear of partner, and experiences of coercive control. At least one affirmative response to the AAS was considered positive for IPV. Eligible women were provided with a description of the study and invited to participate; ineligible women were thanked for their interest.

### Study procedures

Informed written consent was obtained from all participants prior to data collection. The letter of information was read to women who could not read or write, and they were asked to provide a thumb print to indicate consent in lieu of a signature. Data were collected using structured interviews (SI) that consisted of standardized self-reported measures and survey questions designed to elicit information about women’s experiences of IPV, health, resources, and demographic characteristics, which included age, education, employment, income, years of marriage, and number of children. The interviews took, on average, 20 to 40 min to complete and were conducted in a private room at the primary health care center. Safety guidelines were used to guide the process of data collection. To protect women safety, we introduced the study as a women’s health study and we asked the women not to share the nature or the content of the interview with anyone. We did not screen or interview women in front of anyone, including children older than two years of age. Woman who showed signs of distress during the interview, were offered a break and support while maintaining eye contact. At the end of interview, researchers also used a standard debriefing protocol to discuss the signs of a stress reaction and how women could care for themselves if distressed. Women who disclosed suicidal thoughts or behaviors, were assessed using a suicide assessment and if they were at risk of suicide, the attending physician in the center was informed [65]. Some women were linked with the clinic social worker if additional assistance was needed to manage distress or access services. Each woman received a gift certificate of 50 SR from a local grocery store as a token of appreciation. Ethics approval was obtained from the Research Ethics Board at Western University and Institutional Review Board (IRB) for both Ministry of Health and King Abdullah International Medical Research Center (KAIMRC)/MNGHA in Saudi Arabia prior to recruitment.

### Measurement

Data were collected using six established self-report measures, each of which has demonstrated reliability and validity, along with survey questions, to gather demographic information. All measures were originally created and tested by English. In this study, Arabic versions of each scale were used; as described below, these Arabic measures were either tested in previous research or in this study with good evidence of reliability and validity.

#### IPV severity

A 27-item Arabic version of the Composite Abuse Scale [88] was used to measure severity of IPV. The Original 30-item Composite Abuse Scale is a self-report measure of IPV experiences [75] that asks women to rate how often in the past 12 months they experienced abusive actions on a 6-point likert scale ranging from *never* (0) to *daily* (5). Responses to individuals items are summed to produce total scores, and scores for four dimensions of abuse: Severe Combined Abuse, Emotional Abuse, Physical Abuse, and Harassment [89]. The total score is a continuous measure of IPV severity, where higher scores indicate more severe IPV. Hegarty et al. suggest the use of a total score ≥ 3 as the cut-off for case finding in general practice, such that women who score 3 and above are considered to be experiencing abuse [75]. The CAS has been used to measure IPV among clinical populations in different countries [90, 91]. The Arabic version of the CAS was adapted and translated from the English version while retaining the same format as the original CAS [92]. In the initial development, 29 of 30 items was included; one sexual abuse item (*puts foreign objects in my vagina*) was removed because it was seen as inappropriate to the Saudi culture [92]. This and other sexual abuse items have been critique and modified or replaced in other studies conducted in Western contexts [93, 94]. Subsequently, factor analysis of the Arabic CAS revealed that 27 items distinguished four different types of abuse (physical abuse, verbal abuse, sexual abuse and controlling behavior) and demonstrated evidence of validity [88]. Internal consistency reliability of the total score for these 27 items was .903, with subscales demonstrating excellent to good internal consistency (alpha = .78 to .91). Based on these results, the manifest variable severity of IPV was measured in this study using the total score on the 27-item Arabic CAS.

#### Severity of child abuse

The Arabic version of the Childhood Trauma Questionnaire (CTQ) [95] was used in this study to measure severity of child abuse. The CTQ [96] is a 28-item self-report measure that provides brief screening for histories of child abuse. Participants are asked to indicate the frequency of events in 5 areas (i.e. physical, sexual, and emotional abuse, physical and emotional neglect) on a 5-point likert scale ranging from *never* (1) to *very often true* (4). The CTQ has demonstrated strong reliability in different populations [97, 98], including women exposed to IPV [99]. The Arabic version of the CTQ was translated into the Arabic language and pilot tested with a sample of 40 Arab graduate students living in Great Britain by Al-Zahrani [95]. As a result of pilot testing, Al-Zahrani changed the five sexual abuse item to one indirect question (“I was subjected to an immoral situation that has negatively affected my personality”). In this study, the original 5 sexual abuse items were included after translating them to Arabic and conducting a pilot study to test the full scale with a sample of 30 Arab immigrant women living in Canada. In that pilot study, Cronbach’s alpha ranged from 0.87 to 0.92. In this current study, Cronbach’s alpha ranged between 0.78 to 0.85. The latent variable severity of child abuse was constructed using total scores from three CTQ subscales (physical, emotional, and sexual abuse). Items from the 2 neglect subscales were not included because this study is focused on abuse experiences. Neglect is conceptualized as the failure of caretakers to provide for a child’s basic physical and emotional needs (e.g. food, shelter, love, belonging, and support) [100]. However, this failure might not be intentional, but could be link to poverty or other factors that are out of a parent’s control.

#### PTSD symptoms

The Arabic version of PTSD Checklist-Civilian version (PCL-C) [101] was used to measure PTSD symptoms. The original PCL-C [102] is a 17-item self-report summated rating scale that corresponds to the three *DSM-IV* symptom clusters of re-experiencing (5 items), avoidance/numbing (7 items), and hyperarousal (5 items). Respondents are asked how much they have been bothered by each PTSD symptom in the past month on a 5-point severity scale ranging from *not at all* (1) to *extremely* (5). Responses to all items are summed to yield a continuous measure of PTSD symptom severity (range 17–85) with higher score indicating more severe PTSD symptoms. The PCL-C has proven suitable for use in varied populations who have experienced trauma [103, 104], including women who have experienced IPV [105]. In this study, Cronbach’s alpha was 0.89 for the total score. The manifest variable PTSD symptoms was measured using the total the PCL-C continuous score.

#### Depressive symptoms

The Arabic version of 20-item Center for Epidemiologic Studies-Depression (CESD) Scale [106] was used. The CESD [107, 108] is a self-report scale designed to measure depressive symptoms in the general population and reflects the DSM-IV criteria for depression. Respondents are asked to rate the frequency of symptoms experienced in the past week on a 4-point likert scale ranging from *rarely or none* (1) to *most of the time* (4). The most widely used self-report measure of depressive symptoms, the CESD has established reliability and validity with different populations [65, 109]. The Arabic version of the CESD retains the original format and response options [106] and has demonstrated adequate internal consistency (alpha = .84, .88) in studies of Arabic women [106, 110]. Cronbach’s alpha in this study was 0.86. The manifest variable of depressive symptoms was measured using the total CESD score.

#### Chronic pain severity

To measure chronic pain severity, the Arabic version of 7-item Chronic Pain Grade (CPG) scale [111] was used after translating it to Arabic and pilot testing it. The CPG scale measures chronic pain in the past six months [112]. Participants are asked to rate their current pain intensity, worst pain intensity, and average pain intensity in past 6 months on scales ranging from 0 (*no pain*) to 10 (*pain as bad as it could be*). Participants also rated pain-related interference with daily activities, change in ability to take part in activities, and change in ability to work on scales ranging from 0 (*no change*) to 10 (*extreme change*). Using standard scoring, the pain intensity score (0–100) is calculated by multiplying the mean of the three intensity items by 10. Similarly, the pain disability score (0–100) is calculated by multiplying the mean of the three disability items by 10. Four grades of chronic pain can also be derived by combining the number of disability points and pain intensity scores: Grade 0 (pain free); Grade I (low disability, low intensity); Grade II (low disability, high intensity); Grade III (high disability, moderately limiting); Grade IV (high disability, severely limiting). The CPG has demonstrated strong reliability and validity among primary care patients [112], general population [113], and women who have experienced IPV [16, 47]. The pilot testing with Arab immigrant women showed that the internal consistency reliability of CPG was acceptable (.93 for pain intensity and .97 pain disability) [111]. In the current study, internal consistency was 0.87 for the pain intensity scale and 0.91 for the pain disability scale, suggesting adequate internal consistency. In this analysis, the latent variable chronic pain was constructed using the pain intensity and pain-related disability scores to capture the severity of chronic pain.

#### Perceived family support

The Arabic version of family support subscale of the Multidimensional Scale of Perceived Social Support [114] was used to measure perceived family support. The Multidimensional Scale of Perceived Social Support (MSPSS) [115] is a 12-item summated rating scale that measures of the adequacy of perceived social support from family (4 items), friends (4 items) and a significant other (4 items). All items are rated on a 7-point scale ranging from *very strongly disagree* (1) to *very strongly agree* (7). Item responses are summed to produce total and subscale scores, where higher scores indicate greater perceived adequacy of social support. There is evidence of reliability and validity of the MSPSS in different samples [116, 117]. The Arabic version of MSPSS uses a 3-point rating scale (*disagree, neutral, agree*) instead of 7-point rating scale because researchers and cultural experts agreed that Arabs are less likely to use middle response categories when presented with this many options [114]. In this study, only the family subscale score was used because family relationships are seen to be of greater importance than relationships with friends in the Arab world [69]. Internal consistency of the family support scale was 0.865.

### Analysis

Structural equation modeling (SEM) was used to test the hypothesized model. Prior to the main analysis, the extent and magnitude of missing data were explored. Missing data occurred at a low frequency across all scales (range from 0 to 0.7%) and missing completely at random (MCAR, based on a non-significant Little’s test [118]. Therefore, missing values on scales were imputed using the woman’s average score on that scale. This approach is appropriate because people are usually internally consistent across a set of items that form a scale; furthermore, this strategy allowed all cases to be retained for analysis [119].

Descriptive statistics were computed for all manifest variables and indicators of latent variables (Table 1) to assess normality of the distributions. According to Kline [77], data are non-normal if the skewness index (SI) > 3 and kurtosis index (KI) > 10. Descriptive statistics showed that the assumptions of multivariate normality were not met. The correlations among the variables were inspected and suggested no evidence of multicollinearity (Table 2).

**Table 1:** Descriptive statistics for manifest variables and indicators of latent variables (*N* = 299)

Variable	Measure	Mean	SD	Range	Sk	Ku	%Above cut score^a^
Severity of wife abuse	CAS	26.44	19.69	1–127	1.42	2.64	96.7%^b^
Severity of child abuse	CTQ-emotional	9.18	4.48	5–25	1.20	0.88	45.5% (low to severe)
	CTQ-physical	7.48	4.08	5–25	2.12	4.48	30.4% (low to severe)
	CTQ-sexual	6.85	3.59	5–25	2.67	7.76	38.5% (low to severe)
Perceived family support	MSPSS-family	8.45	2.82	4–12	−0.27	−1.22	–
PTSD symptoms	PCL-C	46.72	15.50	17–82	0.05	−0.75	75.6%
Depressive symptoms	CESD	27.63	13.15	0–60	0.18	−.68	78.6%
Chronic pain severity	CPG_intensity	52.39	27.62	0–100	−0.41	−0.61	48.2% (high disability)^c^
	CPG_disability	41.74	31.80	0–100	0.125	− 1.16	

^a^Provided for scales where cut scores have been developed

^b^A cut-off total score of 3 was used, although this may conservatively underestimate IPV as 27 items were included on the Arabic CAS versus 30 on the original scale

^c^Pain grade 3 or 4, consistent with high disability chronic pain

*CAS* Composite Abuse Scale, *CTQ* Childhood Trauma Questionnaire, *MSPSS* Multidimensional Scale of Perceived Social Support, *PCL-C* PTSD checklist-Civilian version, *CESD* Center for Epidemiologic Studies-Depression, *CPG* Chronic Pain Grade, *Sk* Skewness Index, *Ku* Kurtosis Index

**Table 2:** Pearson Correlation among Measured Variables

Measured Variables	1.	2.	3.	4.	5.	6.	7.	8.	9.
1. CAS	1.00								
2. CTQ-emotional	0.186	1.00							
3. CTQ-physical	0.241	0.629	1.00						
4. CTQ-sexual	0.117	0.270	0.399	1.00					
5. MSPSS-family	−0.273	− 0.143	−0.284	− 0.152	1.00				
6. PCL-C	0.449	0.360	0.288	0.130	−0.280	1.00			
7. CESD	0.509	0.277	0.274	0.104	−0.369	0.748	1.00		
8. CPG_intensity	0.260	0.176	0.168	0.004	−0.136	0.466	0.461	1.00	
9. CPG_disability	0.268	0.109	0.212	0.116	−0.171	0.430	0.459	0.667	1.00

*CAS* Composite Abuse Scale, *CTQ* Childhood Trauma Questionnaire, *MSPSS* Multidimensional Scale of Perceived Social Support, *PCL-C* PTSD Checklist-Civilian version, *CESD* Center for Epidemiologic Studies-Depression, *CPG* Chronic Pain Grade

Using MPLUS version 7 [120], the robust maximum likelihood (RML) method was used to correct the standard errors for some non-normality in the data. In the analysis, severity of child abuse and severity of IPV were allowed to correlate. The analysis was run twice with different indicators of mental health problems in each model (i.e. PTSD symptoms in Model 1 and depressive symptoms in Model 2) because PTSD and depressive symptoms are proposed to be distinct reactions to traumatic events and, therefore, might play different mediating roles. The fit indices root-mean-squared error of approximation (RMSEA), standardized root mean squared residual (SRMR), comparative fit index (CFI), Tucker–Lewis Index (TLI), and chi-squared indices were used to assess fit or each model with the data. The critical values for assessing model fit for CFI and TLI are .90 for adequate fit and ≥ .95 for excellent fit. For RMSEA, a value of .08 indicates adequate fit while ≤.06 indicates excellent fit [121]. Standardized path coefficients (*β*) were examined to enable comparisons of paths within models while unstandardized coefficients (B) were used to compare the strength of paths across the models [77]. The standardized path coefficients reflect the “pure” associations between the variables, controlling for other variables in the model. Given the present sample size and the complexity of the model, women’s demographic characteristics were not controlled for and/or included in the models.

## Results

Demographic characteristics of the sample are summarized in Table 3. The mean age of participants was 36.1 years (SD = 10.20, range 18–64), the vast majority of whom (92.3%) were mothers. Women’s educational backgrounds varied widely from those who were unable to read and write (10.9%) to those who had earned a university/college degree. Only 22.7% of women were employed and the mean of their personal income was 1702 Saudi Riyal (SR) per month (SD = 3522 SR). Women’s estimated monthly household incomes ranged from 0 to 53,000 SR with a mean 9455 SR (SD = SR 7324). However, 5.6% of women did not know their husband’s income. Of interest, although all women screened positive for IPV, only 31.4% of women reported that they believed that their husbands were abusive.

**Table 3:** Profile of Demographic Characteristics of the Sample (*N* = 299)

Characteristics	Range	Mean	*SD*	% (n)
Age	18–64	36.1	10.20	–
Years of Marriage	0.08–45	15.0	11.227	–
Polygamous Marriages	–	–	–	18.7 (56)
Mothers	–	–	–	92.3 (276)
Number of children	0–14	4.23	3.021	–
Duration of wife abuse (Years)	0.08–45	11.4	9.985	–
Believed Husbands Abusive	–	–	–	31.8 (95)
Unable to read and write	–	–	–	10.7 (32)
Formal Education				
Elementary school	-	-	-	30.1 (90)
High school	-	-	-	29.1 (87)
Diploma	-	-	-	2.7 (8)
University degree	-	-	-	21.4 (64)
Monthly total household income (SR)^a^	0–53,000	9455	7324	–
Monthly women’s income (SR)^b^	0–23,000	1702	3522	–
Employed	–	–	–	22.7 (68)

^*a*^ *Monthly total household income in CAD (range: 0–18,550; mean: 3309; SD: 2563)*

^*b*^ *Monthly women’s income in CAD (range: 0–8050; mean: 595; SD: 1232)*

As shown in Tables 1, 96.7% of women met the threshold for IPV on the CAS using Hegarty’s guideline (total score ≥ 3 is the cut-off), while between 30.4 and 45.5% of women had experienced abuse as children. Scores on standardized health measures suggest a profile of substantial health problems. Rates of mental health problems were very high, with 75.6% and 78.6%women reporting symptoms consistent with PTSD and depression, respectively. Almost half of women in this sample were living with high disability chronic pain.

### Measurement model

**Fig. 2:**
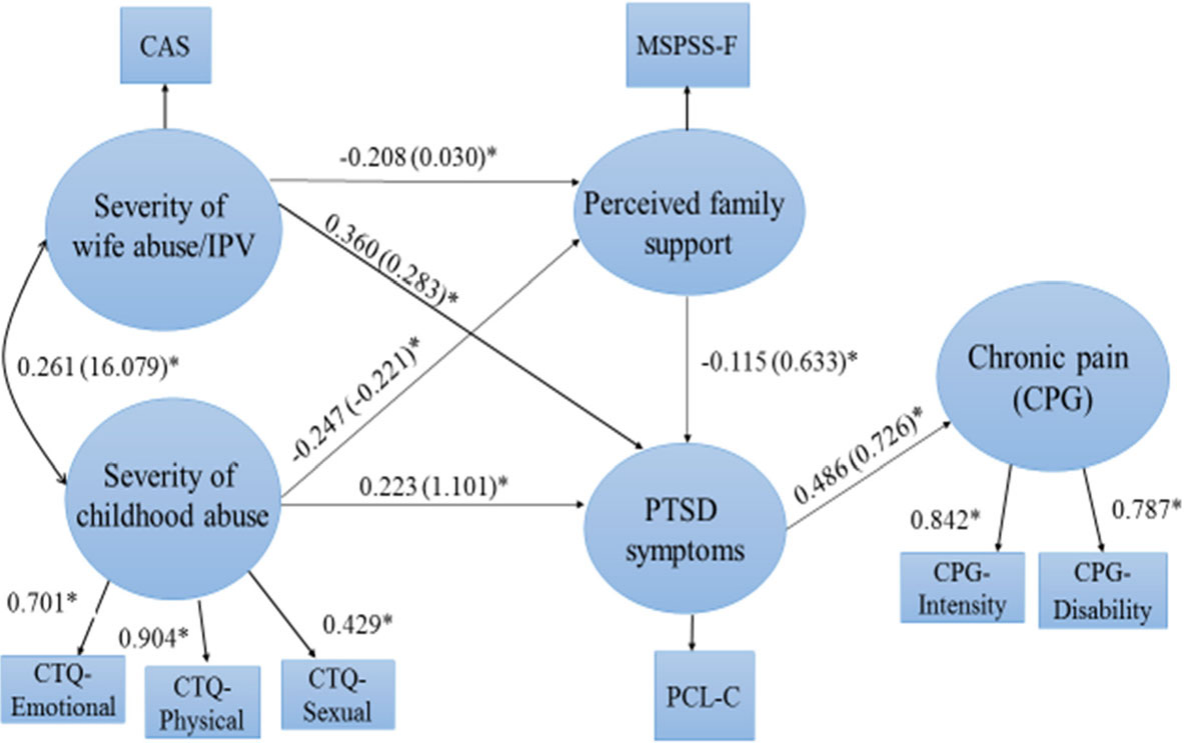
Structural equation model with manifest variables, latent variables, measures, and standardized (unstandardized) path coefficients (Model 1). *P < 0.05

**Fig. 3:**
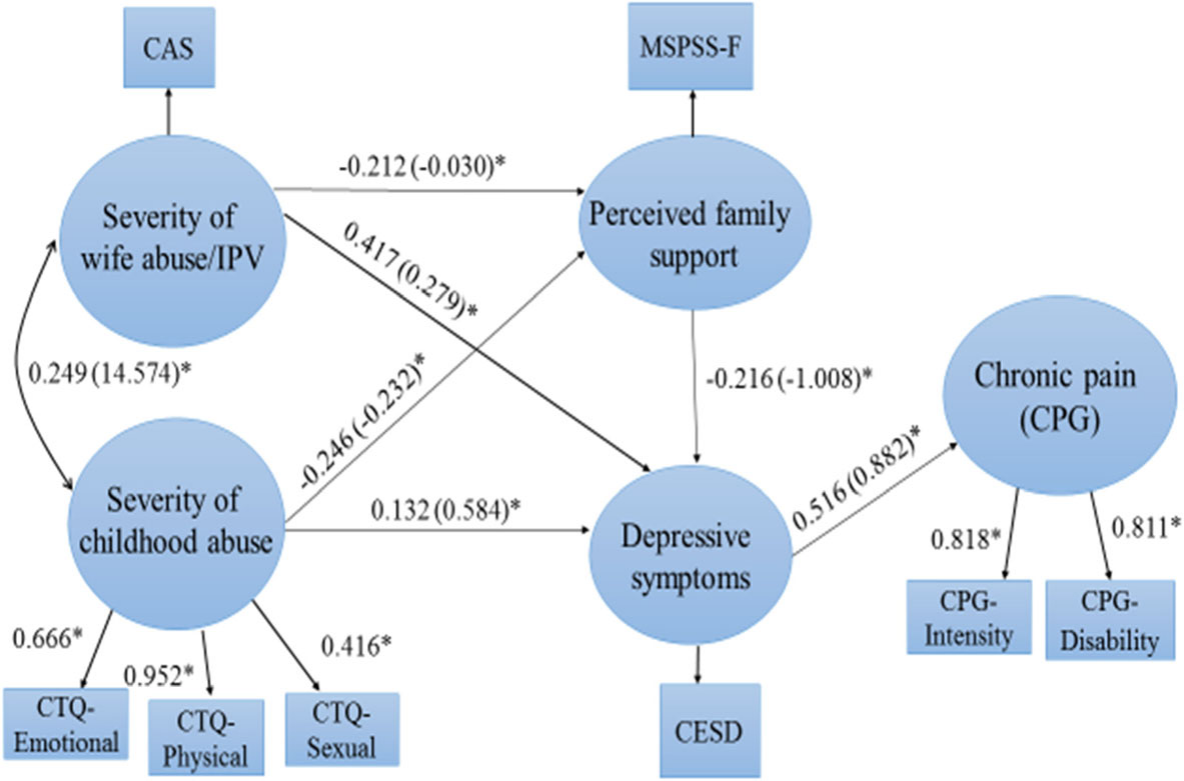
Structural equation model with manifest variables, latent variables, measures, and standardized path coefficients (Model 2) *P < 0.05

Standardized factor loadings for the latent measures (child abuse and chronic pain) were statistically significant and of substantial magnitude (0.416–0.952) (Figs. 2 and 3), providing support for the measurement model. There were no unreasonable parameter estimates, such as negative variances or correlations greater than one, and all appeared to be in the expected range of values.

### Model fit

Using SEM, Model 1 (using PTSD symptoms as an indicator of mental health) was found to adequately fit the data, χ2 (13, *N* = 299) = 37.581 (*P* = 0.003), CFI = 0.957, TLI = 0.907, RMSEA = 0.080 (90% CI: .051–.110), SRMR = 0.027. Model 2 (using depressive symptoms) was an excellent fit with the data, χ^2^ (13, *N* = 299) = 22.653 (*P* = 0.046), CFI = 0.983, TLI = 0.964, RMSEA = 0.050 (90% CI: .007–.083), SRMR = 0.022. The modification indices for both models were below 4 and theoretically unreasonable. Since both proposed models accounted adequately for the observed co-variances among the variables, they were retained without modification.

### Direct versus indirect effects of IPV and child abuse

Model 1 accounted for 27.2% of the variance in PTSD symptoms and 31.6% of the variance in chronic pain severity. Standardized regression coefficients (*β*) and unstandardized coefficients (B) for each path, along with *p* values, are shown in Fig. 2 and Table 4. Neither IPV severity nor child abuse severity exerted direct effects on chronic pain. As hypothesized, but both IPV severity (*β* = 0.360) and child abuse severity (*β* = 0.223) had significant positive direct effects on PTSD symptoms. Furthermore, PTSD symptoms mediated the effects of both IPV severity (*β* = 0.175) and child abuse severity (*β* = 0.108) on chronic pain. In addition, direct negative effects of both IPV severity (*β* = − 0.208) and child abuse severity (*β* = − 0.247) on perceived family support were observed, and perceived family support had a negative direct effect on PTSD symptoms (*β* = − 0.115). However, perceived family support did not mediate the effects of either IPV or child abuse on PTSD symptoms or chronic pain.

**Table 4:** Effect estimates for Model 1 (with PTSD Symptoms)

Structural paths	Unstandardized coefficients	Standardized coefficients	SE	Critical ratio	*P*-Value
Direct effects					
Wife abuse → PTSD symptoms	0.283	0.360	0.045	7.922	0.000^*^
Wife abuse → Perceived support	−0.030	− 0.208	0.063	−3.334	0.001^*^
Wife abuse → Chronic pain	0.123	0.105	0.061	1.707	0.088
Child abuse → PTSD symptoms	1.101	0.223	0.075	2.961	0.003^*^
Child abuse → Perceived support	−0.221	− 0.247	0.063	−3.921	0.000^*^
Child abuse → Chronic pain	0.411	0.056	0.071	0.785	0.432
Perceived support → PTSD symptom	−0.633	− 0.115	0.054	− 2.122	0.034^*^
Perceived support → Chronic pain	−0.060	− 0.007	0.059	− 0.123	0.902
PTSD symptoms → Chronic pain	0.726	0.486	0.064	7.592	0.000^*^
Indirect effects					
Wife abuse → Chronic pain	0.206	0.175	0.032	5.457	0.000^*^
Child abuse → Chronic pain	0.888	0.108	0.040	2.704	0.004^*^
Wife abuse → PTSD symptoms	0.019	0.024	0.014	1.758	0.079
Child abuse → PTSD symptoms	0.140	0.028	0.015	1.834	0.067

*p < .05

Model 2 accounted for 33.2% of the variance in depressive symptoms and 32.4% of the variance in chronic pain severity. Standardized and unstandardized coefficients for each path, along with *p* values, are shown in Fig. 3 and Table 5. While direct effects were not observed between either IPV severity or child abuse severity and chronic pain, both of these variables had direct effects on depressive symptoms (*β* = 0.417, *β* = 0.132, respectively). In addition, depressive symptoms was mediated the effects of both IPV severity (*β* = 0.216) and child abuse severity (*β* = 0.068) on chronic pain. While perceived family support had a direct negative effect on depressive symptoms (*β* = − 0.216), it was also a significant mediator of the effects of both IPV (*β* = 0.046) and child abuse (*β* = 0.053) on depressive symptoms.

**Table 5:** Effect estimates for Model 2 (with Depressive Symptoms)

Structural paths	Unstandardized coefficients	Standardized coefficients	SE	Critical ratio	*P*-Value
Direct effects					
Wife abuse → Depressive symptoms	0.279	0.417	0.049	8.526	0.000^*^
Wife abuse → Perceived support	−0.030	− 0.212	0.062	−3.394	0.001^*^
Wife abuse → Chronic pain	0.078	0.069	0.064	1.069	0.285
Child abuse → Depressive symptoms	0.584	0.132	0.060	2.223	0.026^*^
Child abuse → Perceived support	−0.232	− 0.246	0.059	−4.192	0.000^*^
Child abuse → Chronic pain	0.704	0.093	0.067	1.393	0.164
Perceived support → Depressive symptom	−1.008	−0.216	0.052	−4.121	0.000^*^
Perceived support → Chronic pain	−0.472	− 0.059	0.061	− 0.976	0.329
Depressive symptoms → Chronic pain	0.882	0.516	0.065	7.918	0.000^*^
Indirect effects					
Wife abuse → Chronic pain	0.246	0.216	0.039	5.575	0.000^*^
Child abuse → Chronic pain	0.515	0.068	0.032	2.130	0.033^*^
Wife abuse → Depressive symptoms	0.031	0.046	0.018	2.608	0.009^*^
Child abuse → Depressive symptoms	0.234	0.053	0.019	2.844	0.004^*^

*P < .05

### Comparing model 1 and model 2

In comparing the two models using unstandardized coefficients (B), PTSD and depressive symptoms operated in similar ways as mediators, but the magnitude of effects differed. Both PTSD (B = 0.206) and depressive symptoms (B = 0.426) were significant mediators of the effects of IPV severity on chronic pain severity, but depressive symptoms had a stronger mediating effect than PTSD symptoms. Similarly, PTSD (B = 0.800) and depressive symptoms (B = 0.515) mediated the relationship between child abuse severity and chronic pain severity, yet PTSD symptoms had stronger mediating effect than depressive symptoms. The direct effects of PTSD and depressive symptoms on chronic pain were more consistent (B = 0.726; B = 0.882, respectively), although the effect of depressive symptoms was slightly stronger.

## Discussion

The results of this study make a unique contribution to existing knowledge in several ways. First, these results provide the first evidence about the magnitude of mental health problems and chronic pain among Saudi women and the mechanisms by which abuse is related to chronic pain in this context. Specifically, results of this study reinforce the contribution of lifetime abuse (IPV and child abuse) in predicting the mental health of women who have experienced IPV, and, subsequently to severity of chronic pain. Furthermore, to our knowledge, this is the first study to compare how PTSD and depressive symptoms mediate the relationships between both child abuse and IPV, and chronic pain. The finding that both PTSD and depressive symptoms fully mediated the effects of IPV and child abuse severity on chronic pain in similar ways extends current knowledge about the unique contributions of these mental health problems in the development and continuation of chronic pain.

The finding that PTSD and depressive symptoms mediated the relationship between abuse severity and chronic pain is consistent with the existing literature [16, 46–48]. The relationship of PTSD and depression with chronic pain could be explained by neuro-hormones, neurotransmitters, and inflammatory changes as well as brain alterations that are involved in PTSD and depression and which also play role in pain pathophysiology [39, 122]. In contrast, Tiwari et al. did not find a mediating effect of depressive symptoms and Humphreys et al. found that only depression, not PTSD, predicted chronic pain, although neither study included child abuse in their analyses. Including cumulative experiences of abuse, such as child abuse with IPV in the multivariate analysis might provide a more complex understanding of the actual pathways explaining how women develop health outcomes as a consequence of experiences of abuse [123] by disentangling the unique causal effect of recent and past abuse experiences on health outcomes.

The finding that IPV exerted stronger effects than child abuse on women’s mental health (PTSD and depressive symptoms) can be understood in several ways. These results can be explained by existing evidence that child abuse results in an intense inflammatory response [124] and hyperactivity of the central nervous system; in adulthood, subsequent stress leads to neurobiological vulnerability for the development of stress-related psychiatric disorders [125]. These findings offer further support to the understanding that both distal and proximal abuse experiences can lead to enduring long-term health issues [11]. Collectively, these findings reinforce the importance of including child abuse when studying the health consequences of IPV, since women often experience different types of abuse over their lifetimes with cumulative impacts [12, 13].

This current study contributes to the body of the literature by assessing whether PTSD and depressive symptoms operate in different or similar ways. While both PTSD and depressive symptoms are separate reactions to traumatic events [51, 76], our findings show that, while they both mediate the relationship between abuse and chronic pain, there were differences in the magnitude of effect for each type of abuse. Depressive symptoms was a stronger mediator of the impact of IPV on chronic pain than was PTSD symptoms. In contrast, PTSD symptoms had stronger mediating effect on the relationship between child abuse severity and chronic pain than did depressive symptoms. These findings are noteworthy because they begin to identify the extent to which PTSD and depressive symptoms uniquely shape chronic pain among survivors of child abuse and IPV. On one hand, these findings highlight the independent effects of PTSD and depressive symptoms on the experience of chronic pain, which contradicts the assumption that PTSD and depressive symptoms are a single traumatic stress construct [52, 53]. On the other hand, 74.2% of women in this sample had symptoms consistent with both PTSD and depression based on standard cut-scores on measures of these mental health problems. In the Saudi context, mental health is seen as a social shame, which could be undiagnosed and untreated [126] and this may partly explain high rates of PTSD and depressive symptoms in this current sample. It is possible that the high comorbidity of PTSD and depression in the study sample may explain why PTSD and depressive symptoms operated in similar ways as mediators. Additional research is needed to more fully examine this issue.

The findings that both IPV severity and child abuse severity had significant direct effects on PTSD and depressive symptoms is consistent with a large body of evidence on the mental health consequences of abuse [36, 127]. However, this study’s findings make a unique contribution to existing evidence because the majority of previous studies have not assessed the pathways between abuse and mental health and have focused on one type of abuse (i.e. IPV or child abuse). Furthermore, the study extends this evidence to be inclusive of Saudi women, a population in which the health consequences of IPV have not been previously studied.

In this study, perceived family support mediated the relationship between abuse and depressive symptoms but did not mediate the effect of abuse on PTSD symptoms. This finding reinforces the idea that women’s social resources are crucial in managing their depressive symptoms. It also suggests that different mechanisms may underlie the direct and indirect impacts of abuse on PTSD symptoms versus depressive symptoms. Specifically, perceived family support may be central to depressive symptoms, but not PTSD symptoms. This could be explained by the fact that PTSD symptoms are linked directly to trauma severity [128, 129], while depressive symptoms can be linked to many factors, including social resources [130]. Moreover, PTSD symptoms often go unrecognized by family members and health care providers [131], whereas depressive symptoms are better recognized by as a legitimate health issue, resulting in greater likelihood that family may offer help and support. For example, 51.6% of Canadian women who left abusive partners had symptoms consistent with PTSD but only 7.1% had a diagnosis [20].

Our results show that abuse severity impacts women’s mental health which, in turn, leads to chronic pain, a finding that supports the interaction among physiologic, psychological, and social factors in the production of chronic pain. These findings highlight the interconnection between mind and body, with important implications for how abuse experiences are treated in the health care system. Specifically, our findings underscore the necessity of health care providers understanding the relationships among the severity of abuse, mental health, and chronic pain as a foundation for providing good clinical care. Assessing for abuse experiences as well as identifying and treating PTSD and depression are important strategies that may decrease experiences of chronic pain among IPV and child abuse survivors.

In this current sample, 75.6% and 78.6% of women has symptoms above the cut-off scores for PTSD and depression measures, respectively. In addition, 48.2% of women were living with highly disabling chronic pain. These findings confirm the high prevalence of mental health and chronic pain issues in the current sample. Since there is limited understandings of IPV health consequences in Saudi society, documenting the physical and mental health outcomes of IPV may be useful in raising awareness of these issues among health professionals. As such, result of this study may contribute to recognizing that violence is a health issue worthy of the time and attention of Saudi health professionals. As recommended by the World Health Organization [132], identification of women who have experienced IPV can be achieved using a case-finding approach in which women who are suspected to be victims of abuse (based on risk factors or presentation) are assessed for these experiences. In the context of IPV, effective case finding requires that health professionals have knowledge about risk factors for IPV and the skills to intervene and address health problems and safety issues in ways that are trauma-informed [133]. Yet, future research should examine the effectiveness of a case-finding approach on women’s health, quality of life, and victimization. Treatments for women’s mental health issues found to be effective in the context of IPV, including psychological support and counselling [134], need to be offered to women in health care settings. The finding support the need for primary prevention of abuse and intervention programs should be designed. Thus, health-based interventions need to draw on women’s social networks to help them manage mental health symptoms. There are some promising interventions in this area. For example, a three-phased intervention program for IPV survivors showed a reduction in psychological symptoms and increasing in perceived social support [135]. Positive effects on social isolation and social networking were also found in a group interventions program for women experiencing IPV that used a cognitive-behavioral approach [136]. Social service providers also need to be aware that abused women might experience mental and physical health issues and be prepared to make referrals for health care.

The results of this study should be considered in light of some limitations. This study relied on retrospective self-reports of child abuse and IPV experiences which may result in bias. Self-report measures are often subject to social desirability and people are more likely to minimize their abuse experiences rather than make them up [137, 138], particularly in a context where IPV is a taboo issue. Given the sample size and the complexity of the model that was tested, important factors, including women’s demographic characteristics, personal resources, medications and/or current medical conditions, which might influence health outcomes, were not included in the tested model. Thus, future research needs to consider including demographic characteristics to these models and testing them with larger samples.

Given that chronic pain is a complex biopsychosocial phenomenon, future research should focus on testing explanations about the development and persistent of chronic pain that integrate both biophysical and psychosocial factors. For example, the inclusion of cortisol, an important biomarker for PTSD [139] and depression [140], would benefit future research. As well, measuring pro-inflammatory cytokine and prostaglandin synthesis could provide objective evidence of experiencing pain because they both influence inflammation [141] and the sensitivity of pain receptors [142]. While the use of retrospective data on women’s experiences of child abuse and IPV strengthens the evidence for causal associations between abuse and health outcomes, the cross-sectional study design limits the ability to infer causation to statistical prediction; longitudinal, predictive models are needed to more fully test these and other models. Although a convenience sampling was used, women were recruited from nine primary health care centers located in different neighborhoods, and serving populations with a range of socioeconomic statuses, allowing for greater generalizability of the study findings.

## Conclusion

The finding highlights the significant effects of both IPV and child abuse on women’s mental and physical health. The current study provides evidence through multivariate analysis that both PTSD and depressive symptoms mediate the impacts of IPV severity and child abuse severity on chronic pain severity among women. PTSD and depressive symptoms operate independently as mediators and in similar ways, but with different magnitudes. In addition, perceived family support mediated the relationship between abuse severity and depressive symptoms. These findings underscore the importance of attending to lifetime abuse and depressive and PTSD symptoms, as well as women’s social resources, in chronic pain management and treatment. As well, tailoring health interventions that target women’s social support system and mental health symptoms is important.

## Abbreviations

CAS: Composite Abuse Scale
CESD: Center for Epidemiologic Studies-Depression
CPG: Chronic Pain Grade
CTQ: Childhood Trauma Questionnaire
IPV: Intimate partner violence
MSPSS: Multidimensional Scale of Perceived Social Support.
PCL-C: Posttraumatic stress disorder Checklist-Civilian version
PTSD: Post-traumatic stress disorder
